# Supplementary material for: Duality Vaccination through Nanodesign of Click-Ligated B-Cell Inhibitory Receptor Bridging
Source: bioRxiv. 2026 Oct 30:2026.10.28.762652. Preprint. [Version 1] doi: 10.64898/2026.10.28.762652 (PMC13734137; doi:10.64898/2026.10.28.762652)
Supplement: Supplementary file 1 [file media-1.pdf]

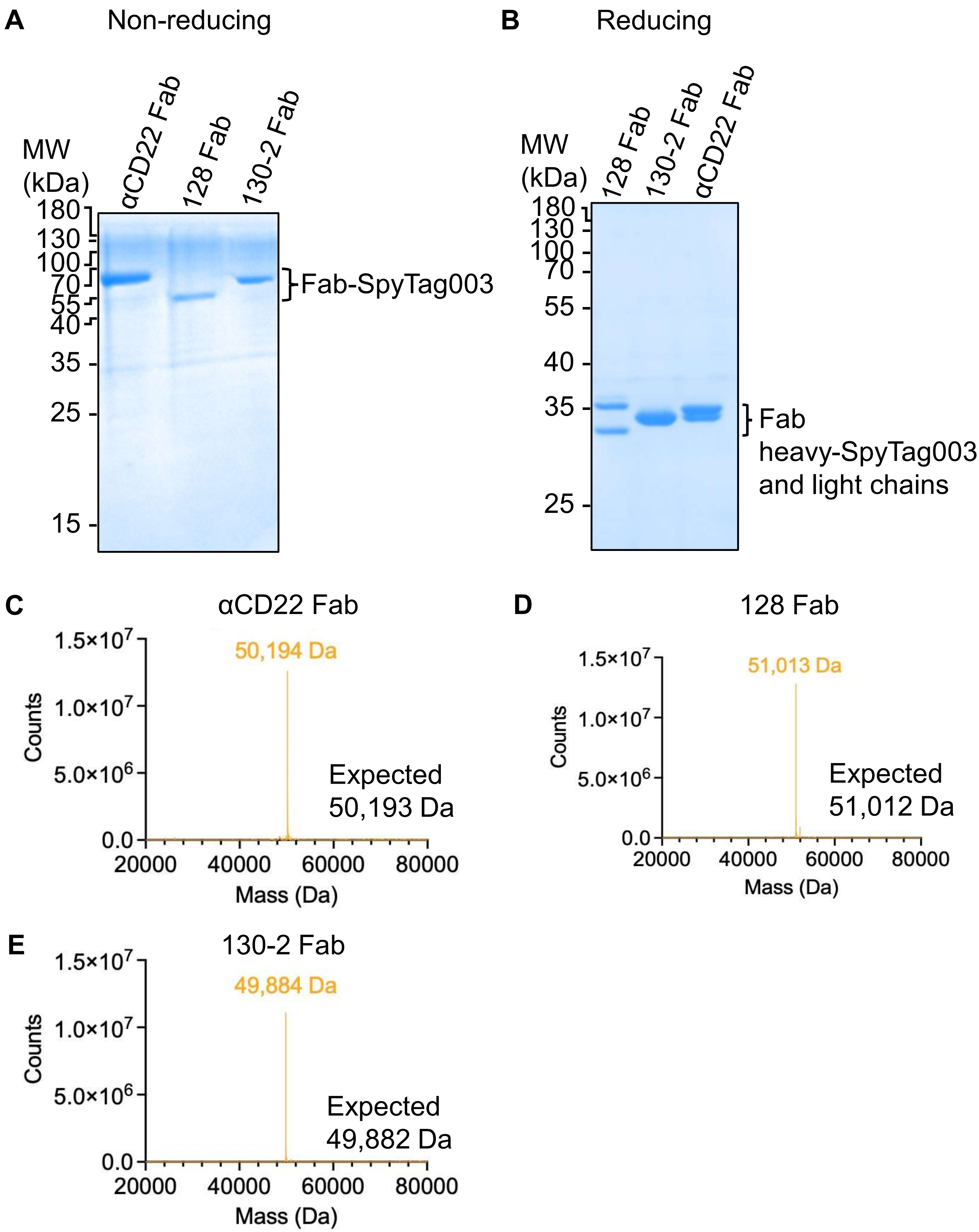

Supplementary Fig. 1. Expression and purification of Fabs to inhibitory receptors. (**A**) Purification of SpyTag003-linked Fabs. Fabs against CD22 or FcγRIIb (128 or 130-2) were purified from Expi293F cells and analyzed by non-reducing SDS-PAGE with Coomassie staining. (**B**) Reducing SDS-PAGE of Fabs as in (A). (**C**) Electrospray Ionization Mass Spectrometry of anti-CD22 Fab, with observed peak mass shown alongside the expected mass. (**D**) Mass as in (C) for anti-FcγRIIb Fab 128. (**E**) Mass as in (C) for anti-FcγRIIb Fab 130-2.

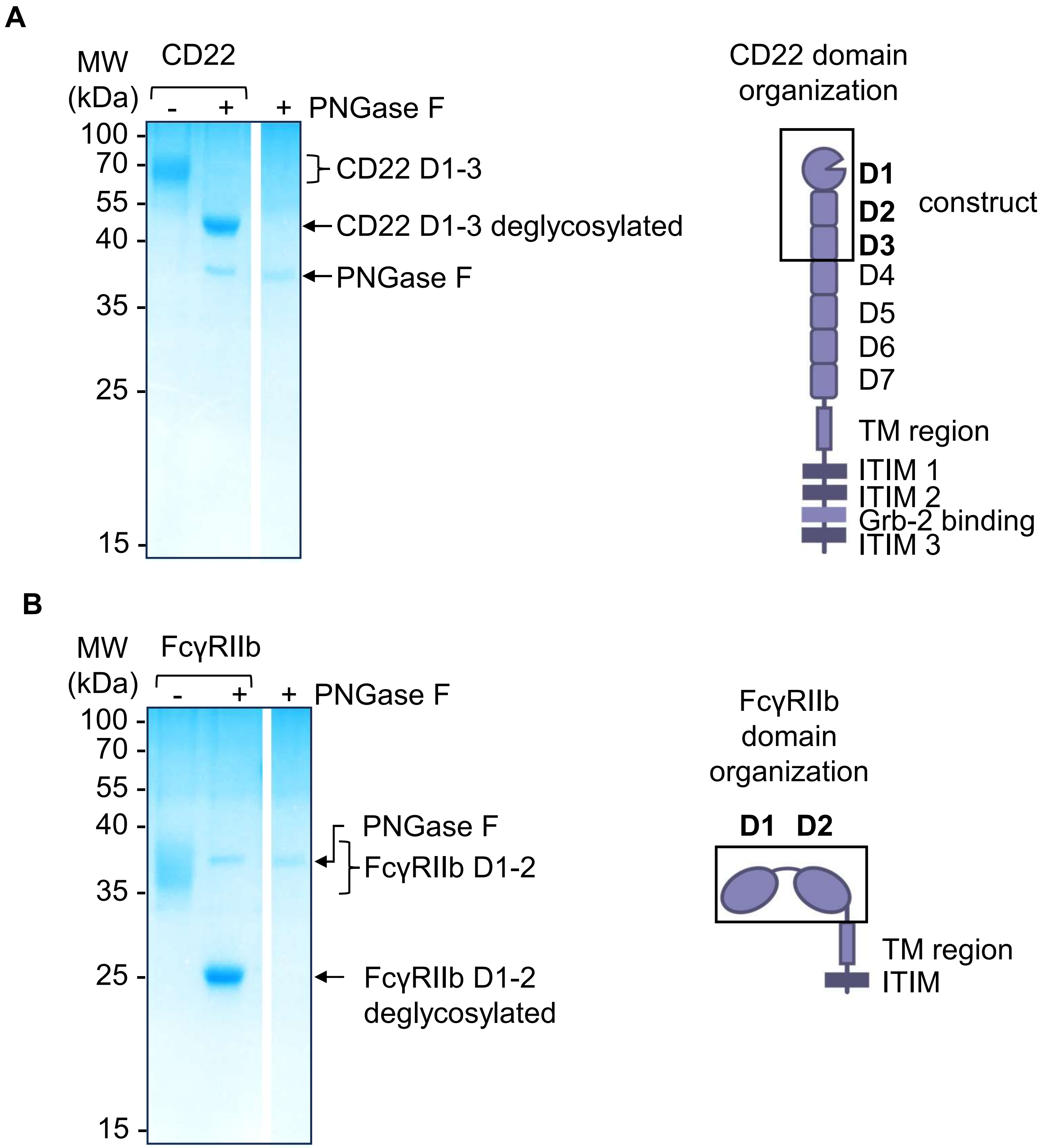

Supplementary Fig. 2. Purification of inhibitory receptor ectodomains. (A) CD22 extracellular domains 1-3 (D1-3) were cloned for expression in Expi293F cells and analyzed by SDS-PAGE with Coomassie staining. **(B)** FcγRIIb extracellular domains 1-2 (D1-2) were cloned for expression in Expi293F cells and analyzed by SDS-PAGE with Coomassie staining. In the lanes without deglycosylation by Peptide:N-glycosidase F (PNGase F), the broad band relates to variable charge from multiple N-linked glycosylation sites.

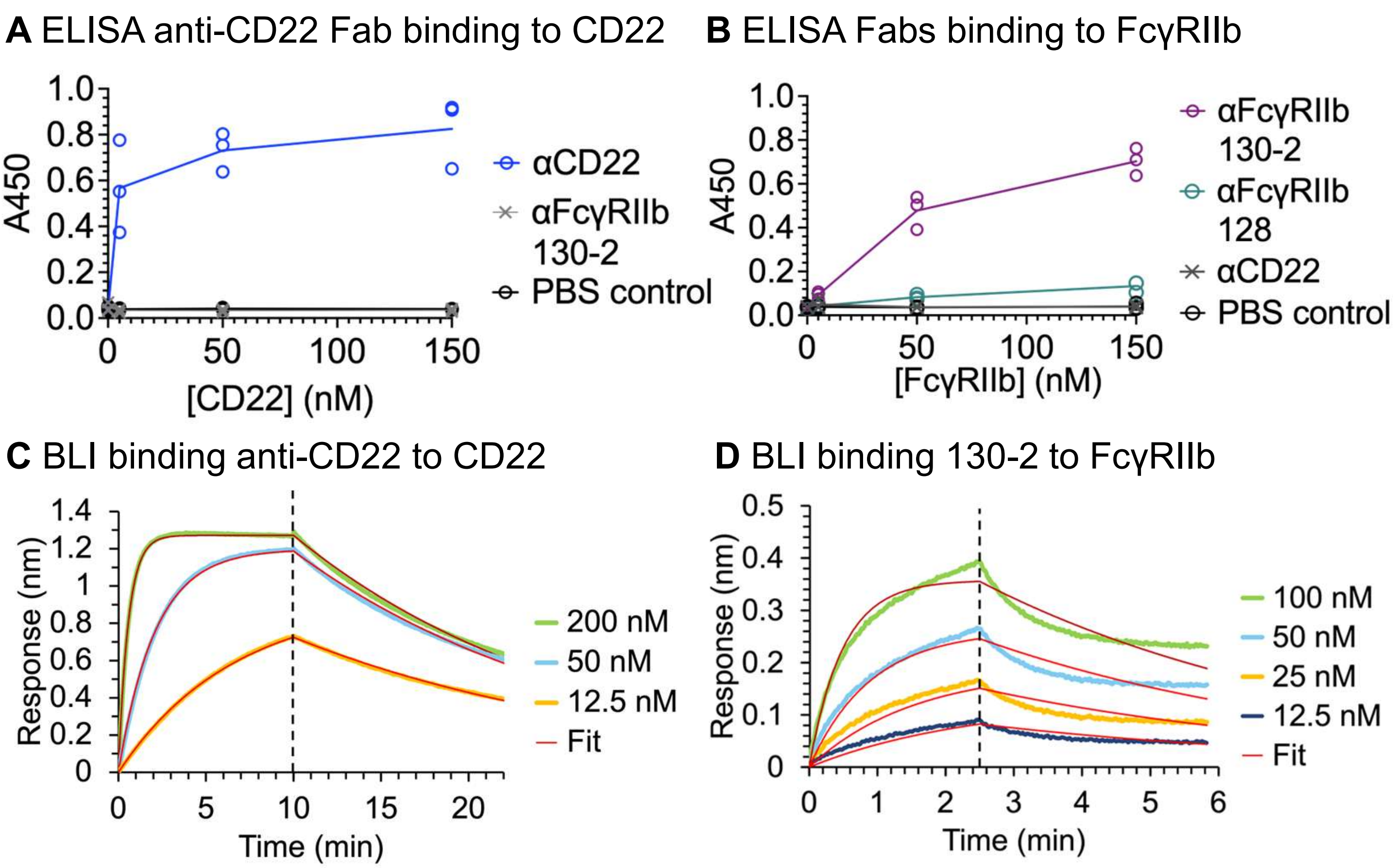

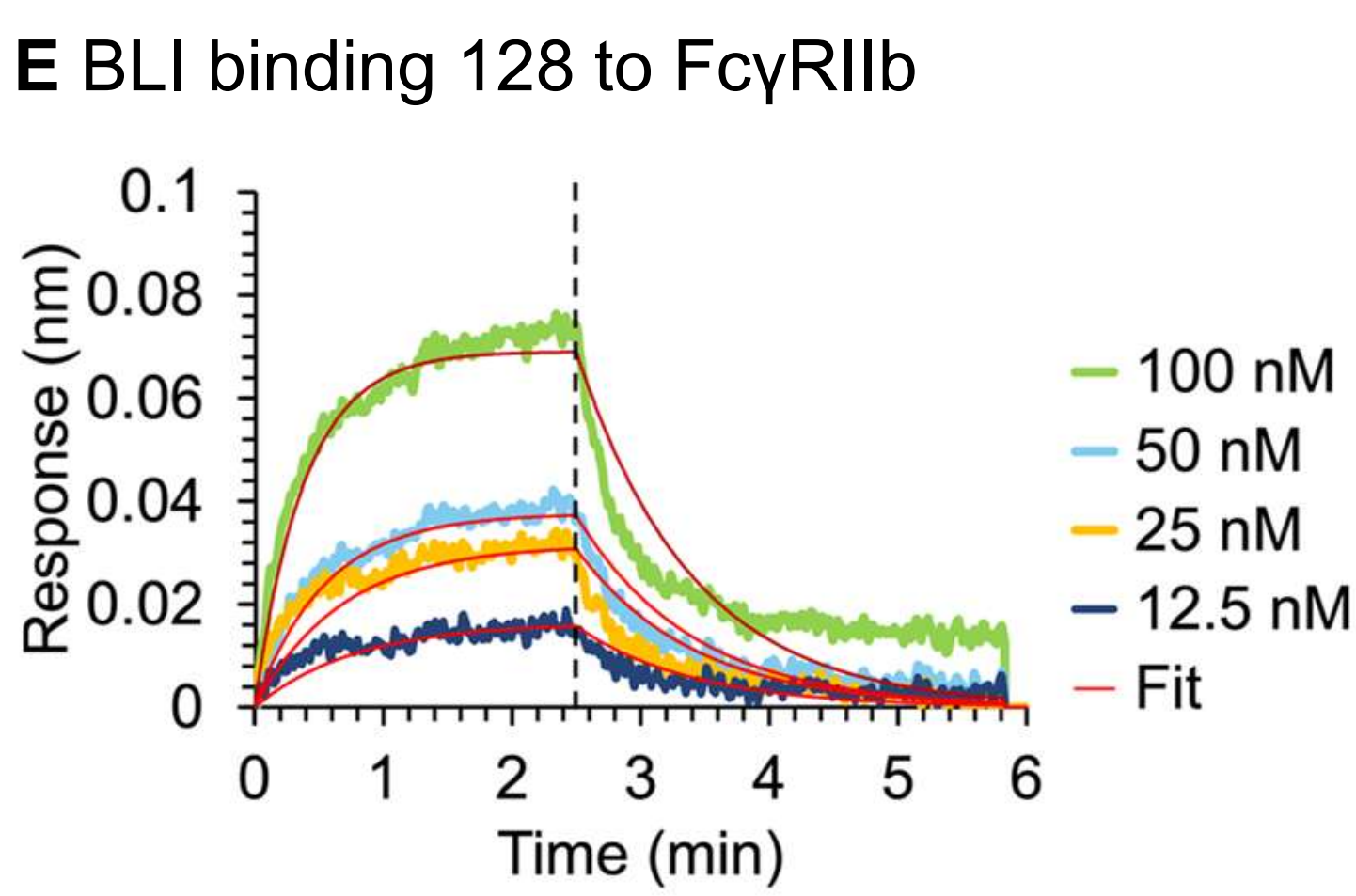

F BLI summary

	K_d (nM)	k_{on} ($M^{-1}s^{-1}$)	k_{off} (s^{-1})
**α-CD22 to CD22**	7.1 ± 0.1	1.4 ± 0.1 × 10^5	9.9 ± 0.1 × 10^{-4}
**130-2 to FcγRIIb**	10 ± 0.3	3.1 ± 0.1 × 10^5	32 ± 0.4 × 10^{-4}
**128 to FcγRIIb**	53 ± 2.8	3.6 ± 0.2 × 10^5	190 ± 2.8 × 10^{-4}

Supplementary Fig. 3. Validation of Fab binding to inhibitory receptor ectodomains. (**A**) ELISA showing anti-CD22 Fab binding to CD22 ectodomain. Anti-CD22 Fab or controls PBS or 130-2 Fab were immobilized on the plate, before varying concentrations of site-specifically biotinylated CD22 ectodomain were added. Detection was then performed colorimetrically using streptavidin-HRP. Each value was plotted for 3 replicate wells with the line connecting the mean. (**B**) ELISA showing anti-FcγRIIb Fab binding to FcγRIIb ectodomain, conducted as in (A). (**C**) BLI of anti-CD22 Fab binding to CD22 ectodomain. Fab was added at 12.5, 50 or 200 nM. Data are overlaid with the model fit in red. (**D**) BLI of 130-2 Fab binding to FcγRIIb ectodomain. Fab was added at 12.5, 25, 50 or 100 nM. Non-ideal binding behavior is likely caused by aggregation at the sensor surface. (**E**) BLI of 128 Fab binding to FcγRIIb ectodomain, as in (D). (**F**) Summary of BLI, showing K_d, k_{on} and k_{off} for Fab binding (mean ± 1 s.d.).

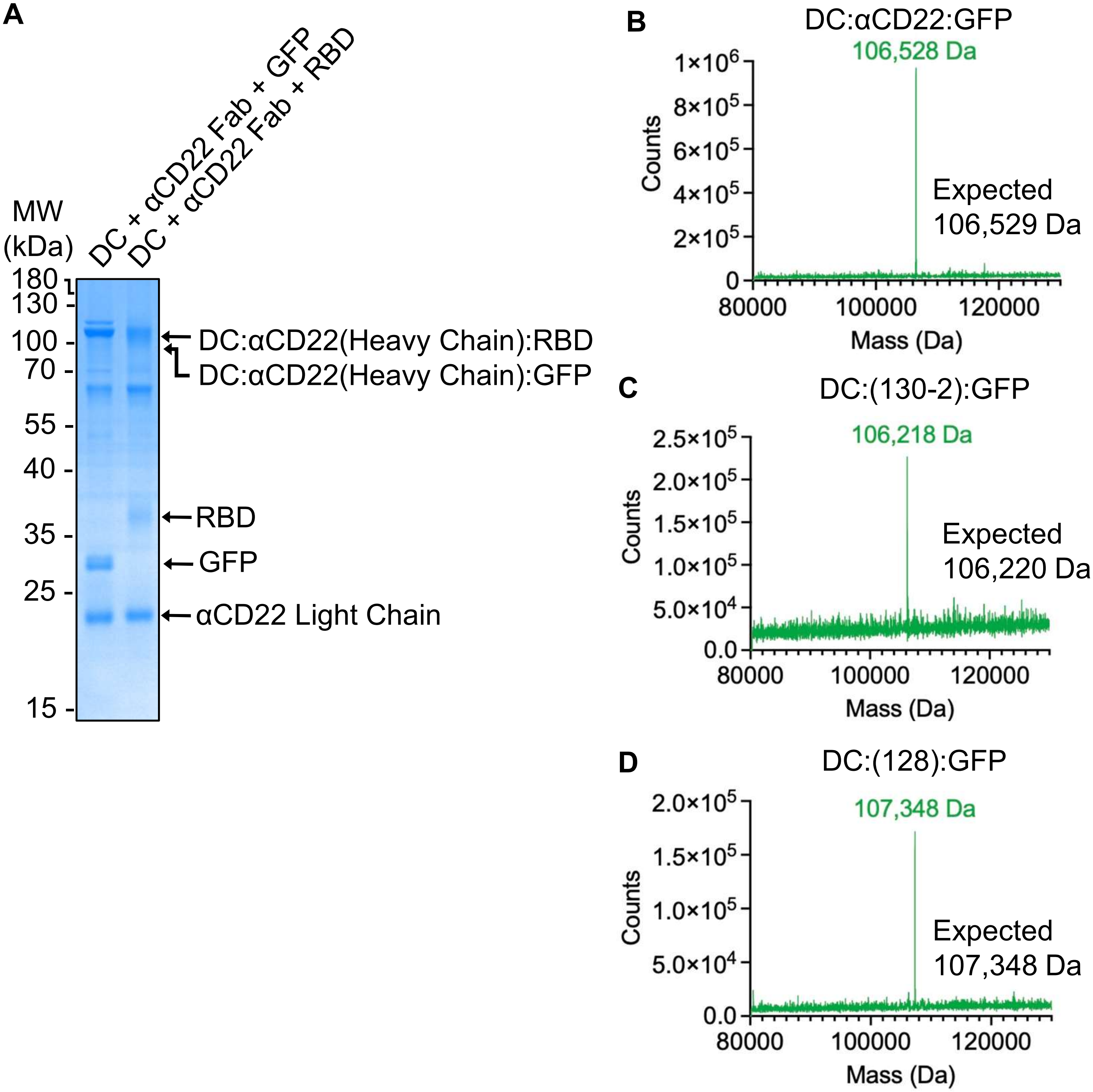

Supplementary Fig. 4. Analysis of inhibitory immunogen assembly. (**A**) Assembly of DoubleCatcher (DC) coupled to anti-CD22 Fab and RBD or GFP, analyzed by reducing SDS-PAGE with Coomassie staining. (**B**) Electrospray Ionization Mass Spectrometry of DoubleCatcher coupled to GFP and anti-CD22 Fab, with observed peak mass shown alongside the expected mass. (**C**) Mass as in (B) for DoubleCatcher coupled to GFP and anti-FcγRIIb Fab 130-2. (**E**) Mass as in (B) for DoubleCatcher coupled to GFP and anti-FcγRIIb Fab 128.

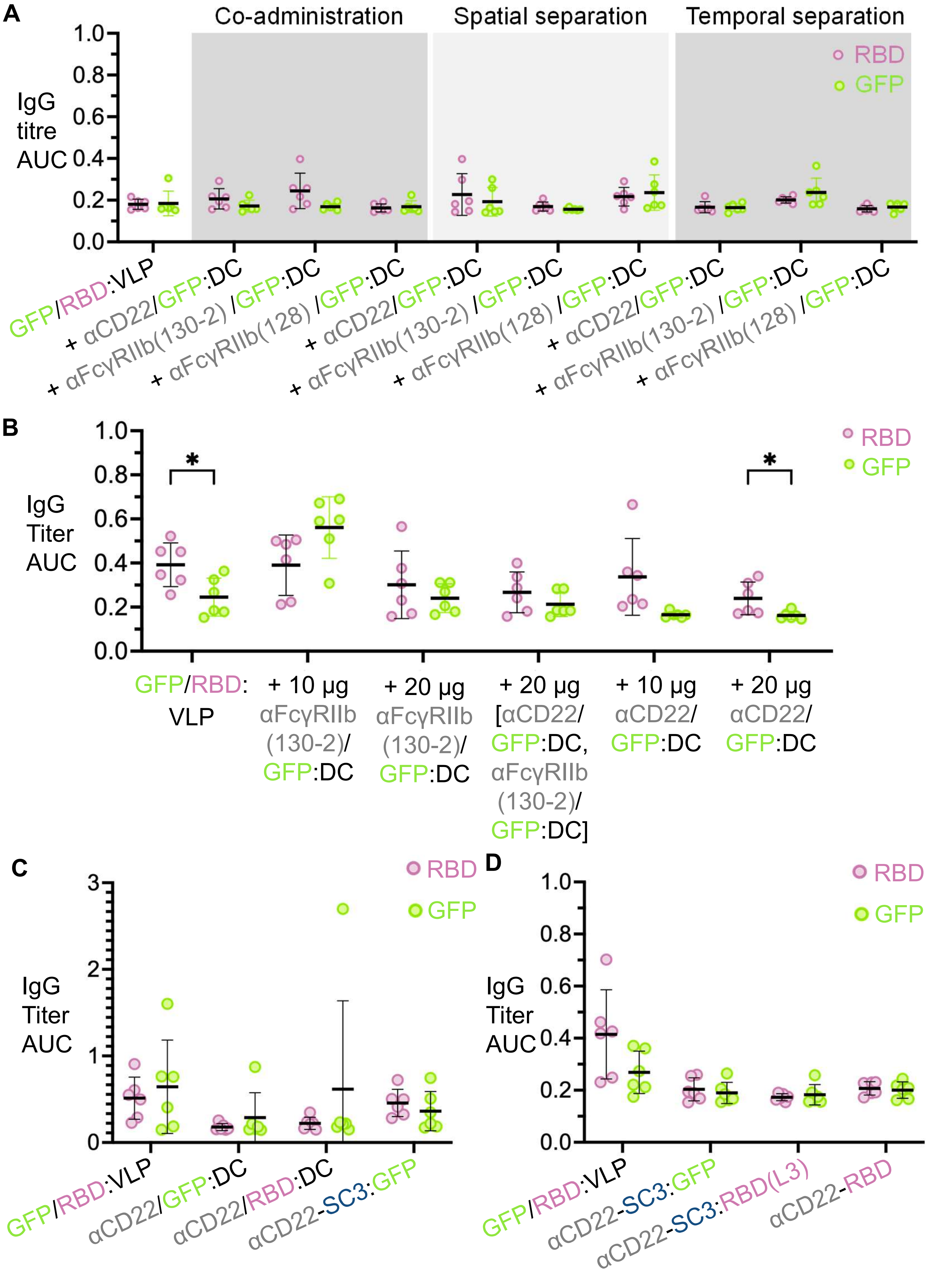

Supplementary Fig. 5 (start)

E

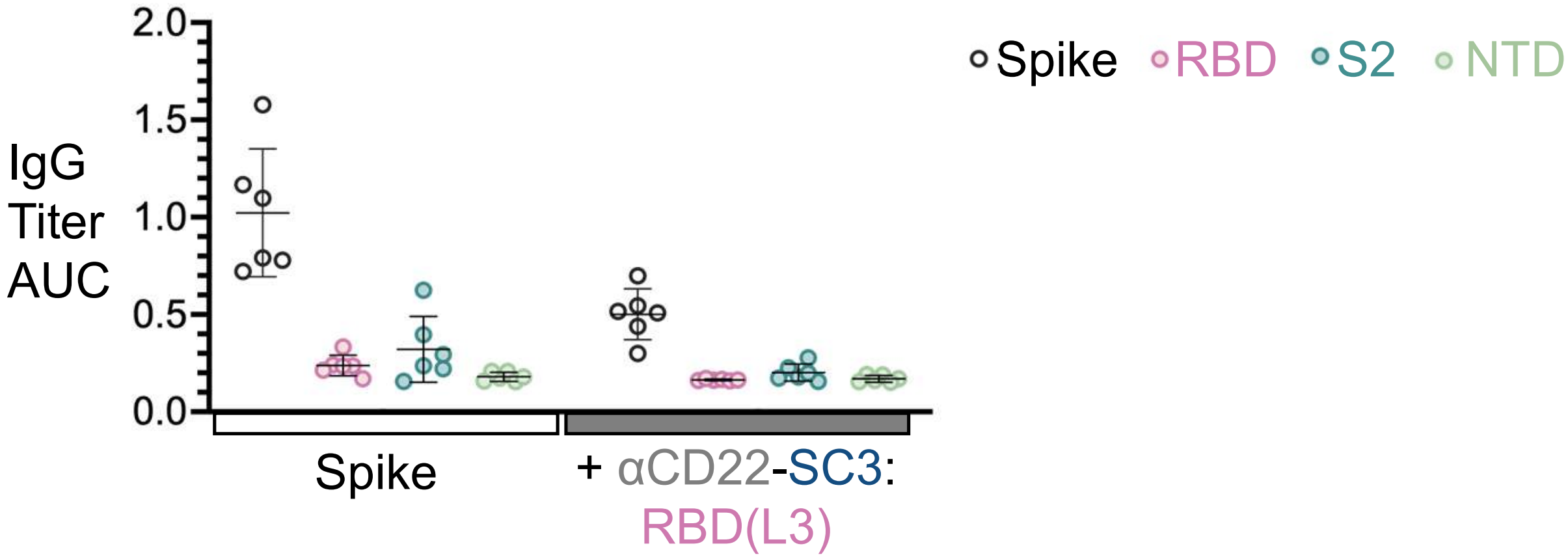

Supplementary Fig. 5. Antibody responses following priming. (**A**) Antibody responses following priming, in line with the boost in Fig. 3C. (**B**) Antibody responses following priming, in line with the boost in Fig. 4B. (**C**) Antibody responses following priming, in line with the boost in Fig. 5B. (**D**) Antibody responses following priming, in line with the boost in Fig. 5G. (**E**) Antibody responses following priming, in line with the boost in Fig. 6E.

A

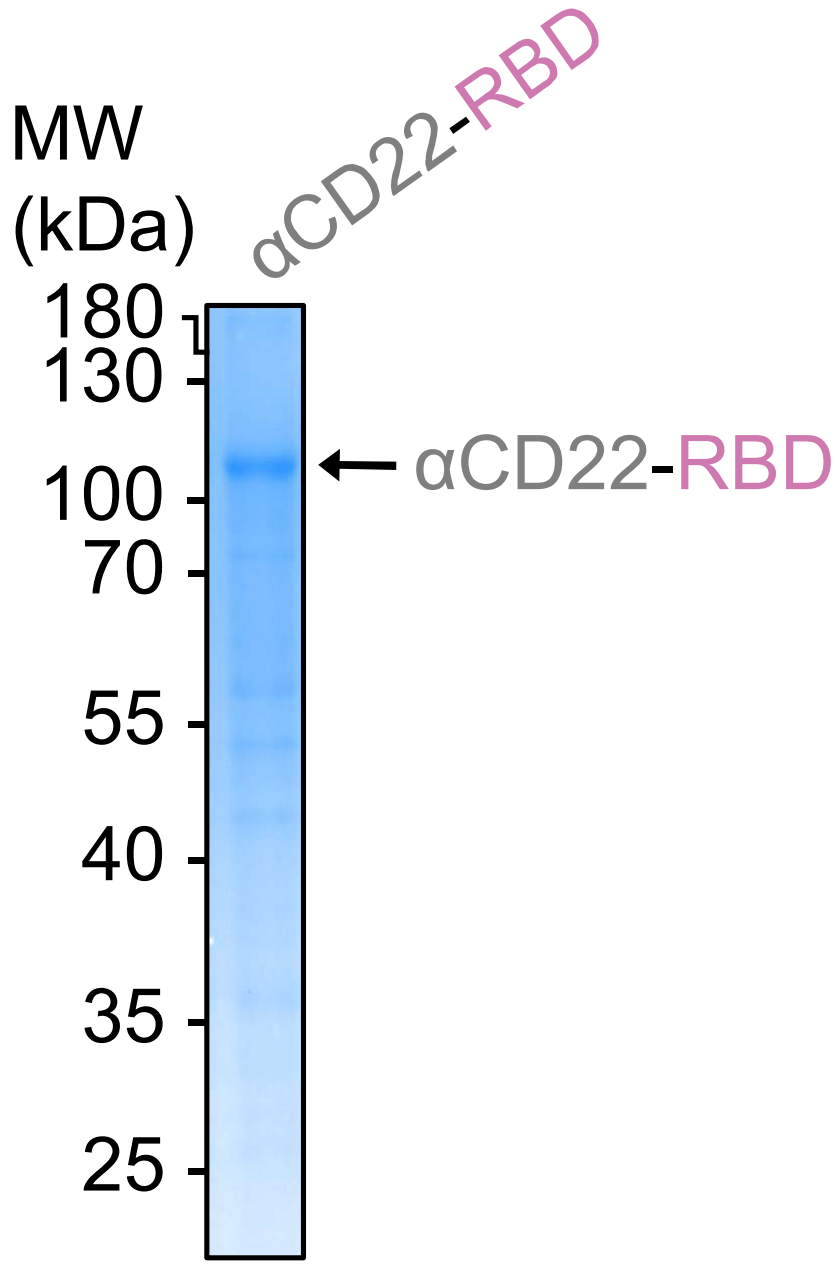

B

Construct	Protein illustration	Total linker length
DoubleCatcher: αCD22:GFP	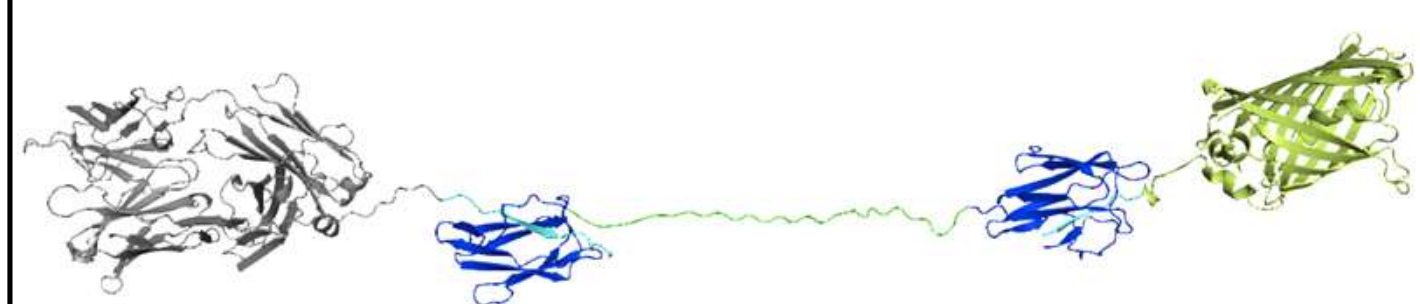	26 aa
αCD22-SpyCatcher003: GFP	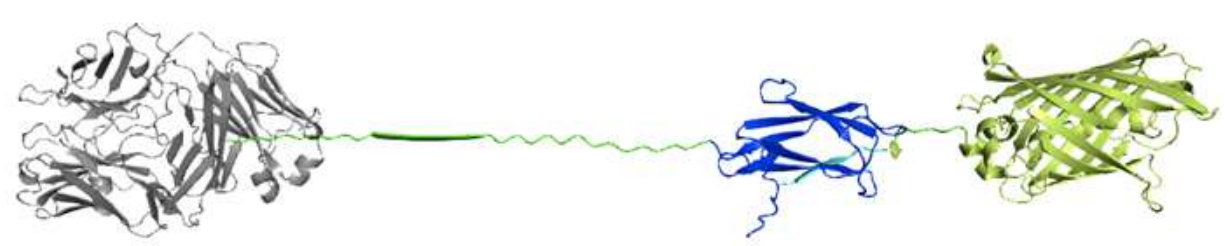	17 aa
DoubleCatcher: αCD22:RBD	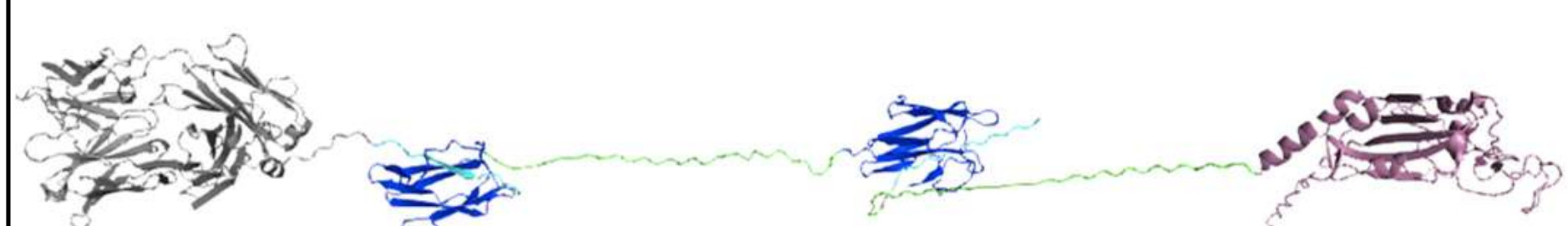	54 aa
αCD22-SpyCatcher003: RBD(L3)	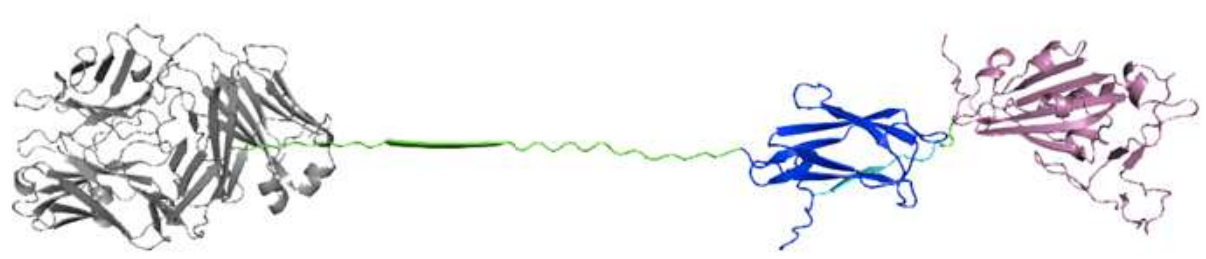	14 aa
αCD22-RBD	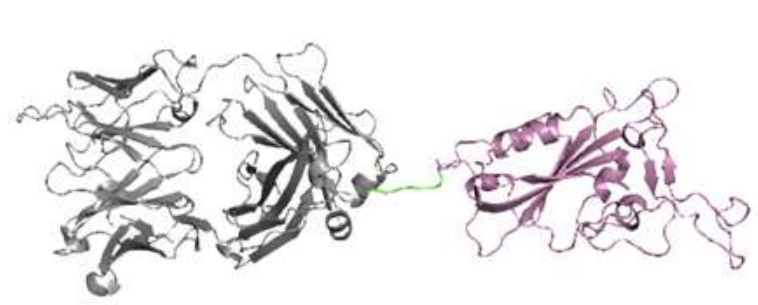	3 aa

Supplementary Fig. 6. Modulating the size of inhibitory immunogens. (**A**) Expression and purification of a direct genetic fusion of anti-CD22 Fab to RBD, analyzed by non-reducing SDS-PAGE with Coomassie staining. (**B**) Illustration of potential extended length of inhibitory immunogens, from AlphaFold prediction of individual units and modeling extended linker conformation. Total linker length is calculated from the Glycine-Serine spacers.

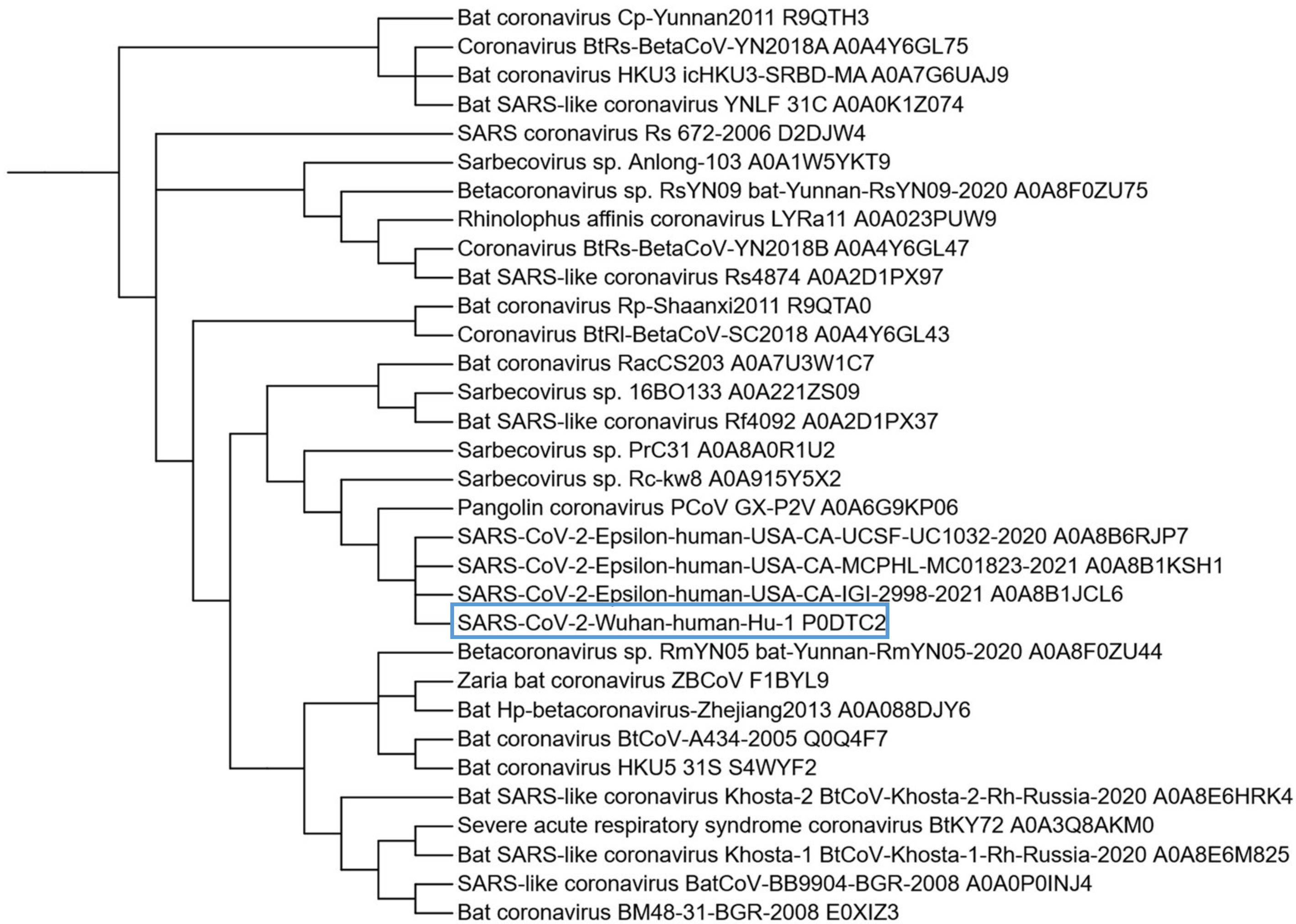

Supplementary Fig. 7. Phylogenetic tree of sequences used in Spike conservation calculation. Labels follow the format Virus_strain-or-isolate, with the trailing code being the UniProt/UniParc accession number for that sequence.
